# Perceptions of frailty pathway implementation in an acute setting among healthcare professionals: a qualitative study using the CFIR 2.0

**DOI:** 10.1186/s12877-026-07683-5

**Published:** 2026-06-03

**Authors:** Rebecca Hui Shan Ong, Jin Wee Ng, Fuyin Li, Gek Kheng Png, Hong Choon Oh, Kiat Sern Goh, Barbara Helen Rosario

**Affiliations:** 1 Health Services Research, SingHealth, Changi General Hospital, Singapore, Singapore; 2 Department of Nursing, Changi General Hospital, Singapore, Singapore; 3 Centre for Population Health Research and Implementation, SingHealth, Singapore, Singapore; 4 Department of Geriatric Medicine, Changi General Hospital, Singapore, Singapore

**Keywords:** Frailty pathway, Acute inpatient setting, Healthcare professionals

## Abstract

**Background:**

Frailty is prevalent among older inpatients and is associated with poor outcomes. Hospital frailty pathways are structured approaches to identify, assess, and manage frailty in acute care, and can potentially improve patient outcomes, but they require adaptation to the local context for successful implementation. This study explored healthcare professionals’ perspectives on implementing a frailty pathway in a tertiary acute-care hospital in Singapore.

**Methods:**

A qualitative study was conducted using focus group discussions with healthcare professionals from diverse disciplines involved in older adult care at a tertiary hospital. Participants were recruited by purposive sampling. The semi-structured topic guide and template thematic analysis were informed by the updated Consolidated Framework for Implementation Research (CFIR 2.0). Findings were presented narratively, organised by CFIR 2.0 domains and constructs.

**Results:**

Seven focus groups (*n* = 41 participants: physicians, nurses, and allied health) were conducted. Facilitators included the perceived credibility and compatibility of the frailty pathway, supportive leadership and teams, tailored staff education, dedicated infrastructure, recognition of frailty as a growing population need, caregiver involvement, community partnerships, interdisciplinary collaboration, and availability of financial resources. Barriers included complex and ambiguous processes, caregiver burden, delirium complicating care, competing clinical priorities, staffing and equipment constraints, sociocultural and health literacy challenges, and financial constraints.

**Conclusions:**

Implementing frailty pathways in acute hospitals requires addressing structural and cultural barriers while leveraging key enablers such as multidisciplinary collaboration, targeted staff training, caregiver involvement, and integration with community care services. Focusing on these factors may enhance uptake of frailty pathways into acute care settings.

**Supplementary Information:**

The online version contains supplementary material available at 10.1186/s12877-026-07683-5.

## Introduction

Globally, ageing populations are transforming healthcare systems, placing increasing pressure on hospitals to manage complex age-related conditions and geriatric syndromes. Among these conditions, frailty has emerged as a critical challenge [1]. Frailty is a multidimensional syndrome characterized by progressive declines in physiological reserve and function, resulting in heightened vulnerability to stressors such as illness, injury, or hospitalisation [2, 3]. In Singapore acute hospitals, frailty affects an estimated 63–81% of older inpatients [4, 5], and is associated with adverse outcomes including prolonged hospitalisation, inpatient mortality, and loss of independence after discharge [4, 6, 7]. These risks have prompted growing interest in frailty pathways: multidisciplinary, evidence-based care processes embedded within hospital workflows to identify, assess, and manage frailty in older adults [8]. Promising evidence supports these pathways: their implementation has been associated with reduced length of stay, lower 30-day readmission rates, less functional decline, and improved one-year survival and likelihood of discharges to home [8–10]. These successes have catalysed international efforts to integrate frailty pathways into routine hospital care [11, 12]. However, evidence also show that implementing frailty pathways is complex, and successful integration depended on understanding local health system structures and resource availability [11, 13].

In Singapore, this work is especially timely as the nation approaches “super-aged” status [14], and older adults already account for 43% of acute hospital admissions [15, 16]. National healthcare strategies such as the Action Plan for Successful Ageing and Healthier SG have emphasised improving frailty care in hospitals [17, 18]. Nevertheless, translating frailty management from policy to practice in the acute setting remains challenging. Prior evidence highlights conceptual ambiguity around frailty definitions, a lack of standardised screening tools, and uneven buy-in across clinical departments as hindrances to implementation [19, 20]. Time constraints, limited access to geriatric-trained professionals, and resource limitations pose further challenges [19, 20].

Locally, Changi General Hospital (CGH) is a 1,000-bed tertiary hospital serving over one million residents in eastern Singapore, and provides a full range of acute inpatient, specialist outpatient, and geriatric services. The campus consisted of a Medical Centre for specialist outpatient services, a Main Building (MB) for acute inpatient care wards, and an Integrated Building (IB) to support acute geriatric and rehabilitative care. Although a unified CGH-wide frailty pathway has not yet been established, multiple frailty-related initiatives are in place. The Clinical Frailty Scale (CFS) [21] is used in the Emergency Department (ED) to identify older adults for frailty interventions, while the IB employs both the CFS and the modified Hospital Frailty Risk Score (mHFRS) [22]. Frailty screening and interventions are also embedded within disease- or service-specific workflows such as the Heart Failure Pathway, Geriatric Surgical Services, and Orthogeriatric Services. An internal multidisciplinary frailty group coordinates frailty education, research, and has developed a frailty flow sheet to facilitate care planning. However, current implementation remains fragmented, with challenges in integrating data flow between the ED and inpatient systems and in extending frailty workflows beyond geriatric specialties.

To better understand these implementation challenges and inform the development of a hospital frailty pathway, we conducted a qualitative study to explore healthcare professionals’ perspectives on strengthening and integrating existing frailty care initiatives at CGH, in anticipation of implementing a hospital-wide frailty pathway in a tertiary acute care hospital in Singapore.

## Methods

### Study design

We conducted a qualitative study using focus group discussions (FGDs). The study was underpinned by an interpretivist paradigm, assuming that reality is socially constructed through individuals’ experiences and contexts. A semi-structured topic guide (Supplementary 1) was developed based on the updated Consolidated Framework for Implementation Research (CFIR 2.0), a theory-informed framework comprising 48 constructs (with 19 sub-constructs) across five domains: Innovation, Outer Setting, Inner Setting, Characteristics of Individuals, and Implementation Process [23]. CFIR 2.0 has been applied in frailty-related implementation research to inform the design, delivery, and scale-up of complex interventions [24, 25]. We used a narrative approach to present the findings, organising themes and sub-themes by CFIR 2.0 domains and constructs. The study is reported in accordance with the Standards for Reporting Qualitative Research (SRQR) guidelines (see Supplementary 2).

### Participants

Using purposive sampling, we recruited doctors, nurses, and allied health professionals aged 21 years or above, with experience in managing older adult inpatients. An advertisement email was circulated to relevant hospital departments to invite staff to participate in the study. Interested individuals were subsequently contacted by the study’s research coordinators, who provided further study details and arranged the focus group discussions. Participants were given time to consider participation before providing written informed consent. Participation was voluntary, and each participant received a SGD $30 token of appreciation for their time.

### Data collection

All sessions were moderated by the first author (and primary coder) using the topic guide. Before each FGD, participants completed a brief questionnaire collecting demographic information, years of work experience, and departmental affiliation. Discussions were audio-recorded with a recorder and transcribed verbatim by a trained research coordinator. Member checking was not conducted due to the time constraints of participants, who were practicing healthcare professionals with limited availability.

### Researcher characteristics and reflexivity

The primary coder is a female Chinese researcher in her 30s with a Master of Public Health and experience in qualitative research. The second coder is a male Chinese researcher in his 20s with an undergraduate background in psychology and experience in qualitative coding. Neither had prior personal relationships with the participants.

### Qualitative data analysis

We employed a template thematic analysis approach, which supports hierarchical coding and offers a structured yet flexible framework for organizing qualitative data [26]. Both deductive and inductive approaches were used: the initial coding template was informed by predefined CFIR 2.0 constructs, while additional codes were developed inductively to capture emerging concepts from the data. An initial template was created from a subset of transcripts and iteratively refined through discussion and comparison of independently coded data. Once consensus was achieved, the remaining transcripts were divided between the two coders and coded using the template. Coding discrepancies were resolved through discussion. All analyses were performed using Dedoose software (version 9.2.22). Data saturation, at which point no new codes or themes emerged that would substantively alter the coding template was achieved by the seventh FGD.

## Results

### Participant characteristics

**Fig. 1:**
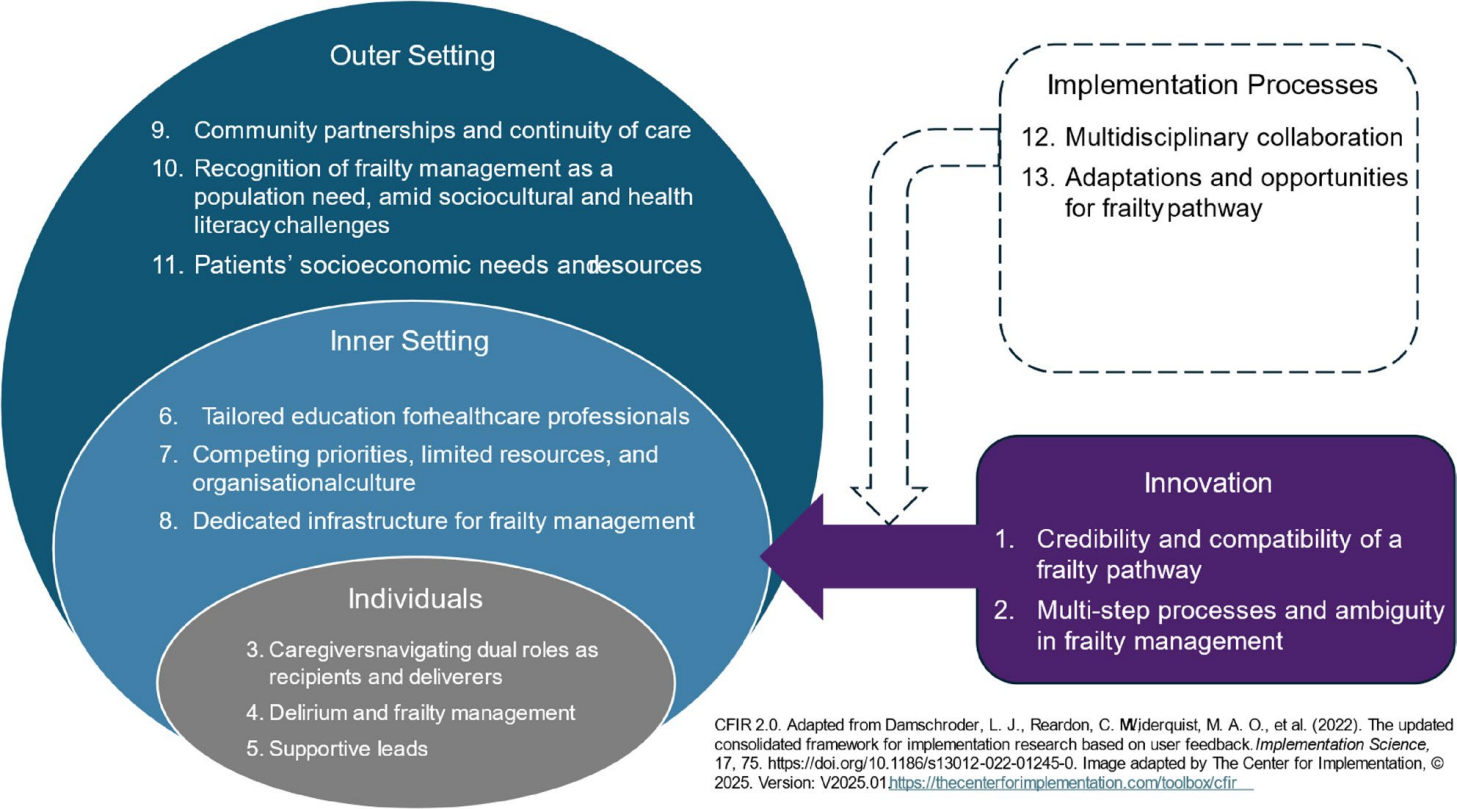
Thematic findings mapped to CFIR 2.0 domains

Seven FGDs were conducted between March and May 2024. Each FGD included 3–8 participants and lasted 75–105 min. A total of 41 healthcare professionals participated in the study. The mean participant age was 35.8 years, and the majority were female (36 participants, 87.8%). On average, participants had 11.2 years of clinical experience (see Table 1). Figure 1 presents an overview of the qualitative themes.

**Table 1:** Participants’ characteristics

Variables	
Total Participants, n	41
Gender, n (%)	
Male	5 (12.2)
Female	36 (87.8)
Mean Age, mean (SD)	35.8 (11.6)
Years of Experience, mean (SD)	10.9 (10.7)
Role, n (%)	
Allied Health	14 (34.1)
Nursing	13 (31.7)
Physician	14 (34.7)
Department, n (%)	
Emergency department	4 (9.8)
General medicine	4 (9.8)
Gastrology	1 (2.4)
Infectious disease	1 (2.4)
Cardiology	1 (2.4)
Psychiatry medicine	2 (4.9)
Wards, community nursing and case management	10 (24.4)
Surgery & anesthesia	4 (9.8)
Allied Health	
Dietetics	2 (4.9)
Medical social services	2 (4.9)
Pharmacy	2 (4.9)
Therapy services	8 (19.4)

## Domain 1: innovation

This theme reflects participants’ views on the frailty pathway’s credibility, its fit within existing workflows, and the challenges of implementing it in complex care environments.

### Theme 1.1: credibility and compatibility of a frailty pathway

**(**CFIR 2.0 Construct(s): Innovation evidence-base; Innovation design; Innovation source.)

Participants perceived frailty pathways as a credible, evidence-informed initiative that aligned with existing hospital workflows. Participants also pointed to pre-existing geriatric services as indicators of institutional readiness for a frailty pathway. Many noted that they were already using validated frailty-related assessments in daily practice, which helped integrate the pathway conceptually:

*“When it comes to the general wards*,* I think there are a lot of things that we look out for to ensure how frail they are. We monitor the fall scoring*,* the Morse fall scale and also the Braden scale to see how it is.” – ID25*,* Nurse*,* FGD4*.

### Theme 1.2: multi-step processes and ambiguity in frailty management

(CFIR 2.0 Construct(s): Innovation complexity.)

Despite the perceived value of a frailty pathway, participants described current frailty management processes as complex and difficult to navigate. Multi-step workflows, unclear referral and care transitions pathways, and structural inefficiencies were commonly cited barriers. As one participant highlighted:

*I really think that managing frailty is not just a doctor-patient relationship though […] Obviously*,* it has to have various things to be plugged into it for the patient’s journey in a way*,* it’s multi-factor. It’s very difficult to just say it’s just a one-to-one thing. It’s a whole community and various things have to fit in. That’s why it’s so difficult. Whatever model you choose*,* it’s really difficult. – ID38*,* Doctor*,* FGD6*.

## Domain 2: individuals

This theme highlights the roles, experiences, and needs of key individuals involved in frailty care, including caregivers, patients, and healthcare providers. Participants discussed how personal circumstances and professional support shaped the delivery and uptake of frailty management.

### Theme 2.1: caregivers navigating dual roles as recipients and deliverers

(CFIR 2.0 Construct(s): Innovation deliverers; Innovation recipients.)

Participants viewed caregivers as essential facilitators in frailty pathways, often acting both as extensions of the care team and as recipients of support themselves. Active family involvement was seen as essential for patients’ care plans:

*“If family members are able to you know like start to walk the patient*,* if the patient is allowed to walk*,* at least patient still mobilise. So*,* the deconditioning chance is like low.” – ID30*,* Physiotherapist*,* FGD 5*.

However, many participants also observed that caregiver stress is not adequately addressed in the current system. They noted that such ongoing strain among caregivers, may, in turn influence care decisions and outcomes.

*“There’s very little emphasis and support for caregiver stress. I think some community hospitals have helped by giving them like 2-week admissions… respite care just for caregiver to take a break. But even that two weeks*,* you still see the caregiver coming every day to visit… because they feel like you know*,* I’ve been taking care of them*,* I feel guilty if I don’t come.” – ID05*,* Doctor*,* FGD1*.

### Theme 2.2: delirium and frailty management

(CFIR 2.0 Construct(s): Innovation recipients.)

Delirium was frequently mentioned as a complicating factor that serves as a barrier to effective frailty management in the hospital. Participants noted that delirium is common among frail inpatients and make care planning more challenging. It also disrupts coordination among the multidisciplinary team and general ward operations:

*“Because (of delirium) the social workers cannot plan because you don’t know what their final stage is going to be like then the physios cannot engage then you cannot engage*,* medical team also frustrated. The team so busy already trying to do all… Yeah*,* it’s very hard to deal with it. I think we all struggle. That’s why we need Geriatrics. (laughter from all)” - ID07*,* Doctor*,* FGD1*.

### Theme 2.3: supportive leads

(CFIR 2.0 Construct(s): Implementation leads; Implementation team members.

Participants emphasized that supportive leadership, was a key facilitator for implementing frailty care. They noted that senior leaders were influential in shaping departmental culture and priorities. They shared that when senior leaders actively endorsed frailty initiatives and modelled a positive attitude, it helped shift mindsets and build momentum for change:

*“Maybe I’m blessed with a team of people*,* especially senior (lead) people who are embracing this (frailty care practices). Who sets the role model for the rest of the department. I mean*,* I’m blessed with a good team of people who really pushes for it. So*,* initially*,* me and my other colleague had a lot of scepticism that […] you cannot do anything one; they’re that old and that*,* right? But as we do more*,* people will*,* eh*,* actually*,* it’s not so bad and then when we send the elderly for a surgery*,* they are so well-polished that anaesthesia is now easier to do. But I think we need to start from the top also. The leaders of the department must have that passion for our patients*,* including the elderly.”* – *ID41*,* Doctor*,* FGD7*.

## Domain 3: inner setting

This theme reflects how organisational factors within the hospital shaped the implementation of frailty care. Participants highlighted both enabling conditions, such as access to education and dedicated facilities, and barriers, including competing priorities and limited resources.

### Theme 3.1: tailored education for healthcare professionals

(CFIR 2.0 Construct(s): Access to knowledge and information; Compatibility.)

Access to knowledge and information was identified as a key facilitator for implementing a frailty pathway. Participants generally understood frailty in terms of physical decline and ageing; they agreed that education was important to improve readiness for a frailty pathway:

*“Educational content is useful because most of our patients are geriatric patients […] so it will be good if we have resources to study.” – ID26*,* Nurse*,* FGD4*.

Participants stressed that education needs to be relevant and targeted. They mentioned that training tailored to specific departments or professional roles would be better received. Without customization, educational efforts might risked disengagement:

*“Make it relevant to them (the healthcare professional). Make it relevant to the physician*,* why do I need to be bothered about frailty. That kind of stuff. […] If it makes sense*,* if it helps make my care better*,* why would I not want to know. Correct. It makes me a better doctor but it’s just that sometimes we might not see it.” – ID 39*,* Doctor*,* FGD7*.

### Theme 3.2: competing priorities, limited resources, and organisational culture

(CFIR 2.0 Construct(s): Relative priority; Culture; Compatibility.)

Participants acknowledged that while frailty management is clinically important, it is often deprioritised amid the many competing demands in acute care. They described how frontline staff were frequently overextended, with limited bandwidth to address frailty on top of urgent medical issues:

*“Because there’s a lot of competing priorities*,* right? For the staff’s time and energy and mental*,* I mean the focus because as it is*,* in a way*,* in the healthcare system*,* that’s already very burdened. People tend to want to prioritise and sometimes things like frailty might not be something that they think of first.” – ID39*,* Doctor*,* FGD7*.

Some suggested that frailty care might be more feasible and effective after hospital discharge, given the time and complexity required.

*“ID38 [Doctor]*: *So*,* as an inpatient aspect*,* I don’t think management of frailty can play a big role*,* but post-hospital discharge is where you actually can intervene […]*.

*“ID34 [Doctor]*: *I would agree. It should be managed in the community because it’s going to take time. Acute admissions are for acute issues.” – FGD6*.

Participants also noted aspects of hospital culture and bureaucracy that hinder frailty care. A risk-averse culture, administrative documentation requirements (e.g. incident reporting for falls), and increasing workload were all mentioned as challenges:

*“Truth is the workload for us is higher and higher […] and definitely also nowadays like*,* patient more elderly and more complex also.” – ID30*,* Physiotherapist*,* FGD5*.

### Theme 3.3: dedicated infrastructure for frailty management

(CFIR 2.0 Construct(s): Structural characteristics; Available resources; Culture.)

Participants frequently cited the IB as a key structural facilitator for frailty management at CGH. They highlighted the IB’s acute wards purpose-built features that supported patient safety and rehabilitation:

*“Integrated Building (IB) is definitely more spacious. Like the walkways*,* the space in between beds is also a lot. So*,* is quite huge. So it is definitely easier for them to do rehab compared to the wards. – ID31*,* Nurse*,* FGD5*.

According to some participants, the care culture in the IB was also viewed as more person-centred, with healthcare professionals having more time to deliver focused frailty care.

*“I just noticed that the nurses in IB wards*,* I think they’re a bit more patient […] the approach that they use*,* it’s really more like person-centred in that sense […] because I think the main building (ward) is also very acute*,* and the nurses have to rush around.” – ID09*,* Occupational therapist*,* FGD2*.

Equipment availability was another facilitator, the IB was perceived as better resourced with equipment for frail patients:

*“ I heard in IB*,* they actually have more Geri-chairs*,* like one patient has one Geri-chair.” – ID30*,* Physiotherapist*,* FGD 5*.

## Domain 4: outer setting

This theme captures how external factors beyond the hospital influenced the implementation of frailty care. Participants discussed gaps in continuity between hospital and community services, low health literacy, and cultural attitudes that affected engagement with frailty pathways.

### Theme 4.1: community partnerships and continuity of care

(CFIR 2.0 Construct(s): Partnerships & connections; Local conditions.)

Participants emphasised that frailty management requires sustained support beyond the hospital. They viewed acute care as important for addressing immediate issues but emphasised that longer-term follow-up in the community was needed for frailty care. However, participants noted several gaps in the transition from hospital to community care, such as limited communication and awareness of community frailty services, and delays in coordination:

*I think frailty is a chronic disease which needs to be managed long term in the community and primary care [….]. It’s also about continuity of care because as I said*,* inpatient once we settled the inpatient thing*,* cleared for home […]*,* there are services (for frailty) that are out there (in the community). Like*,* especially in primary care. So*,* from hospital to home. I find that not many people utilise that even though it’s available. I think if we can find some way to coordinate a bit better with them*,* if they can tell us what are the target patients they think would benefit from their services the most*,* then we know how to utilise them a bit more. – ID34*,* Doctor*,* FGD6*.

### Theme 4.2: recognition of frailty management as a population need, amid sociocultural and health literacy challenges

(CFIR 2.0 Construct(s): Local conditions; Local attitudes.)

Participants acknowledged that frailty is an increasingly significant issue in Singapore, mentioning the rapidly ageing population and the need for sustainable care approaches:

*“Yeah*,* because I think facing an aging population. […] We will have patients who are living ever longer*,* but with more comorbidities and issues and more frail. So*,* I also wanted to know*,* is there a better way to actually manage their conditions.” – ID12*,* Speech Therapist*,* FGD3*.

Despite this recognition, limited health literacy around frailty, among both the public and healthcare professionals, was identified as a barrier to implementing a frailty pathway:

*“You just talk about the general public*,* talk to them about sarcopenia or frailty right*,* I don’t think many people have a good understanding of it.” – ID07*,* Doctor*,* FGD 1*.

Participants suggested that older adults’ attitudes and motivations might play a role. Some observed that many older patients have a passive mindset regarding their own health management, which could hinder engagement in frailty interventions:

*“A lot of our patients also don’t have a lot of ownership of their health… our older patients also still have a very dependent mindset*,* meaning they don’t really understand their health problems.” – ID14*,* Doctor*,* FGD 3*.

Participants highlighted that sociocultural norms influenced how older adults accepted frailty-related support. They shared how factors such as cultural beliefs, family dynamics, and local preferences appeared to shape how patients perceived or participated in certain care initiatives.

*“I think the other part is like cultural sensitivities as well. Like sometime maybe offer daycare to one Malay family maybe the nenek (grandmother) will just feel uncomfortable because like (the) husband.at home does not like (her) to mingle with other people*,* like sitting just beside the pakcik (another unrelated older man).” – ID06*,* Medical Social Worker*,* FGD 1*.

*“I guess it’s also the mindset because as Asians right*,* our elderly don’t like to drink milk. They’ll tell you I have diarrhea whole day long*,* no matter how many times we drill into them*,* right is lactose-free right*,* they will try and then they’ll tell the same thing.” - ID03*,* Dietitian*,* FGD 1*.

Encouragingly, participants described local adaptations and adjustments that improved acceptance of frailty-related nutritional interventions:

*“Kudos to the dietitians who bring in other (milk) flavours. […] Now got chocolate*,* strawberry*,* coffee […] I think this has definitely helped.” – ID05*,* Doctor*,* FGD 1*.

### Theme 4.3: patients’ socioeconomic needs and resources

(CFIR 2.0 Construct(s): Local conditions; Financing.)

Participants highlighted that Singapore’s tiered healthcare financing system helps ensure that essential care is accessible even to patients of lower socioeconomic status, for example, through subsidised medications and financial assistance funds:

*“Certain medications that is under the subsidised price*,* they are relatively cheap. So try to use all these options first then if not*,* there is like MAF*,* Medication Assistance Fund*,* so in which they are also subsidised by the government. Then if all these fails*,* then there is also this MAF Plus*,* Medication Assistance Fund Plus*,* whereby is a*,* I think the government gives some money to the hospital. In which to subsidise patients who*,* want to use expensive medications that is not under all these schemes… So I guess a patient will not be deprived of the medication they need just because they are not able to afford it.” – ID32*,* Pharmacist*,* FGD5*.

At the same time, participants acknowledged that socioeconomic status could influence access to additional supportive services that aid recovery, such as hiring extra caregiving help or affording nutritional supplements, as these participants discussed:

*“ID07 [Doctor]*: *I think I think one thing that is very practical is cost. […] Meat like because we always talk about as you get older*,* you need proper protein*,* you want good protein. It’s expensive.*

ID06 [Medical Social Worker: *Is true. It’s like we will advise the caregiver*,* oh actually it’s very simple*,* our downstairs (first floor of hospital) we will sell this kind of convenient food but is so expensive. […] I think it’s about SGD6 for one. Yeah*,* it’s very easy*,* they already chop nicely for you*,* fragrant and chili already put nicely. You just put in the microwave and heat up. It’s very convenient*,* but it’s very expensive.*

*ID01 [Medical Social worker]: And then they (older adults) need milk feed also. Milk feeds are expensive, just like one small bottle is 3, 4 (Singapore) dollars. Yeah, and one day need 4 bottles.” – FGD1.*

## Domain 5: implementation process

This theme focuses on how frailty pathways were operationalised through teamwork, communication, and adaptive planning. Participants described strong multidisciplinary collaboration within the hospital, while also highlighting opportunities to enhance the frailty pathway.

### Theme 5.1: multidisciplinary collaboration

(CFIR 2.0 Construct(s): Teaming; Engaging innovation deliverers; Assessing context.)

Participants highlighted the importance of a multidisciplinary approach in facilitating the management of frailty, recognising that no single professional group could effectively address the complex needs of frail older adults.

*“These days*,* right*,* to get a very optimal outcome of patients*,* you need a multidisciplinary team. You cannot work alone. No way. You need a multidisciplinary team.” – ID38*,* Doctor*,* FGD6*.

They described a culture of mutual respect and knowledge-sharing at the hospital that enabled effective team-based care. They shared that different professionals were empowered to flag patient-related issues:

*“Speaking from the medical point of view*,* the medical doctors are very supportive of inputs from the nurses. So*,* I mean if we know that the patient is frail and having delirium*,* the nurses will actually highlight it to the doctors and most of the time*,* they take actions.” – ID25*,* Nurse*,* FGD4*.

Participants shared how geriatricians were important clinical leaders and champions for frailty initiatives. They were supported by multidisciplinary teams (nurses, pharmacists, therapists, medical social workers, etc.), forming a group of internal champions whom participants trusted as sources of guidance on frailty care:

*“You need a physician who knows about the ins and outs. I suppose the reason why geriatricians are (important) is because elderly patients usually are frail. So*,* they are the advocate for elderly patients. […] So*,* you need a medical person there to guide their management*,* their medical management; and then all the allied health*,* including nursing*,* from that point of view. Pharmacists with their medication. Dietitian with their nutrition and physiotherapists and speech*,* in terms of their swallowing and physical activity.”* – *ID38*,* Doctor*,* FGD6*.

Participants also described how communication was supported by the hospital’s IT systems and digital platforms (such as electronic medical records and Microsoft Teams), which helped various disciplines stay informed on patient issues and coordinate care:

*“We will look at the (electronic clinical) notes of the physiotherapist*,* occupational therapist*,* dietitian*,* then we will ensure they are taking the supplements that the dietitian recommended*,* and then transfers*,* all that*,* we will also take a look.” – ID26*,* Nurse*,* FGD4*.

### Theme 5.2: adaptations and opportunities for frailty pathway

(CFIR 2.0 Construct(s): Tailoring strategies; Planning; Adapting; Engaging.)

Participants proposed several strategies to guide the future development and integration of a frailty pathway within hospital practice. One common suggestion was to strengthen internal communication and awareness about frailty initiatives across department. Many participants noted limited visibility of ongoing efforts within the hospital, which could hinder coordination and collective readiness for a structured frailty pathway:

*“So*,* I guess one thing is to make things known to people like what is being done because in the first place. […] For me*,* I don’t even know what A&E do*,* in term of how to identify frailty”. – ID32*,* Pharmacist*,* FGD5*.

Another key recommendation was to initiate frailty screening earlier in a patient’s hospital journey, specifically in the ED. Early identification of frail patients was seen as crucial to trigger timely interventions downstream:

*“Should start (frailty assessments and screening) from ED and then also follow up in the wards also. […] Because there will be some changes to patient*,* maybe when they are in ED*,* they are active*,* and then when they come to inpatient*,* they might have some changes in mental capacity*,* or maybe they might have the functional decline. So at least you can follow up on the patient*,* you see the progress.” – ID19*,* Nurse*,* FGD3*.

Participants noted that the absence of standardised protocols for frailty management across different departments might hinder consistent care in the patient’s journey. They advocated for structured, department-specific guidelines so that frailty is managed coherently throughout the hospital stay, including during transitions to community care. They also suggested strategies such as ward-based frailty huddles, incorporating frailty screening into routine workflows, embedding geriatricians or geriatric resource nurses within teams, and adding frailty assessment triggers or decision-support tools into electronic clinical systems.

## Discussion

This study provides insights into hospital-based healthcare professionals’ perspective on implementing a frailty management pathway in an acute care setting. While much of the existing literature originates from Western health systems, our findings highlight context-specific considerations unique to Singapore’s multicultural, tiered-financing, and family-centric care environment. Participants acknowledged frailty as an important issue affecting older patients and supported having a structured frailty pathway with routine screening and targeted interventions. However, translating this intention into practice can be challenging due to various structural, cultural, and workforce-related barriers. Competing clinical priorities, time pressures, staffing and equipment shortages were commonly cited obstacles that require sustained effort to address. Others, such as unclear care processes might be more amenable to improvement. Strengthening the visibility and consistent use of existing tools, such as the frailty flow sheet, alongside developing clearer institutional guidelines, may help promote a more structured and standardised approach to frailty care, improve care coordination and consistency in frailty management, and patient outcomes [27].

Some participants viewed frailty as a lower priority than immediate acute medical issues. This perception stemmed from factors such as the urgency of other clinical demands, skepticism about the effectiveness of frailty interventions in the hospital, and limited familiarity with frailty concepts among staff. These perspectives highlight the need to raise awareness and develop a shared understanding of frailty across the workforce. Engagement with new care models is shaped by healthcare providers’ knowledge, confidence, and underlying beliefs [28, 29]. Structured educational initiatives, such as hospital-wide or department-specific workshops, case discussions, and department-specific training materials, could address knowledge gaps and reinforce the relevance of frailty care in all departments, thereby strengthening the organisational readiness for a future unified frailty pathway [30]. Additionally, delirium was frequently mentioned by participants as complicating the care of frail patients. Given its close overlap with frailty, delirium management cannot be overlooked in the design of a future frailty pathway. At CGH, existing initiatives to build delirium awareness and strengthen clinical capability have shown encouraging outcomes [31, 32]. These efforts represent important groundwork that can be further built upon and integrated into a future frailty pathway.

Although Singapore’s tiered healthcare financing ensures equitable access to essential care, participants noted that patients with greater financial means often secured better support for recovery. This aligns with international evidence that socioeconomic inequalities can impact health outcomes in older adults with frailty [33, 34]. Locally, such disparities suggest the need for social risk screening and proactive linkage to community partners, for instance, embedding referrals to social workers or primary care within the pathway itself. In addition, cultural norms surrounding frailty and ageing, coupled with low health literacy among some patients and families, can undermine adherence to pathway initiatives. Addressing these barriers may require culturally attuned communication strategies and co-developed educational materials to enhance self-efficacy and participation in care [35]. Low health literacy among older patients was noted as another barrier. Since limited health literacy is associated with worse frailty outcomes [36, 37], patient and caregiver education and empowerment should be part of implementing a frailty pathway [38].

Multidisciplinary collaboration emerged as a consistent enabler of frailty management and will be critical for pathway implementation. Regular communication, shared goals, and embedding geriatric expertise in clinical teams were seen as enablers that could help normalize frailty management in the future unified frailty pathway. These measures have also been noted in other contexts to promote shared ownership of frailty care [39, 40]. Participants further emphasised that sustaining frailty management required hospital-to-community linkages. Timely discharge planning, coordinated handovers, and ensuring patients have access to community rehabilitation, social support services, and primary care follow-up were all deemed critical. Establishing interoperable data systems, dedicated transitional care pathways, and sustained multidisciplinary teamwork across settings are strategies supported in the literature to improve these care continuities [41, 42].

Our findings highlight the value ofpurpose-designed environments and infrastructure for frailty care. Participants believed the design of CGH’s Integrated IB, with wider spaces, rehabilitation zones contributed to better functional outcomes and safety for frail patients, which aligns with evidence that built environments can impact patient outcomes [43]. This setting also fostered a more patient-centred care culture, making it easier to integrate current and future frailty initiatives into daily routines. While not all settings have such dedicated buildings, adopting similar design principles from the IB (e.g., mobility-friendly layouts, therapy spaces) could help other hospitals adapt frailty-friendly environments even within existing infrastructure [44, 45].

Finally, this study highlights the dual role of caregivers, as facilitators of frailty care and as individuals at risk of burnout. In Singapore, families provide the bulk of care for older adults [46]. Caregiver involvement was cited a key facilitator of frailty management within and beyond the acute care setting. They often bridge gaps in care plan adherence, nutrition, home modifications, and polypharmacy medication management, especially among cognitively impaired or low-literacy patients [47–49].

However, the psychological and logistical strain they face can undermine the sustainability of frailty interventions, which is also documented in other studies [19, 50]. It may be beneficial for the future frailty pathway to integrate caregiver-focused interventions to help alleviate this burden and improve the overall success of frailty care plans. Emerging evidence suggests that psychological interventions such as cognitive behavioural therapy and mindfulness-based approaches can reduce depressive symptoms among caregivers of older adults [51], and group-based support or technology-enabled coaching can also be effective [51]. Engaging and supporting caregivers should be considered central to frailty pathway implementation. Future efforts could involve co-designing frailty care models with input from patients and their families to ensure the programs are practical, acceptable, and culturally appropriate.

### Limitations

This study was conducted in a single tertiary hospital in Singapore, which may limit the generalisability of the findings to other settings. Although purposive sampling across multiple disciplines and the use of the CFIR 2.0 framework provided a comprehensive understanding of context-specific implementation challenges, the study relied only on the perspectives of healthcare professionals. Additionally, employing the same framework to both guide data collection and analysis could potentially constrain the analyses, as pre-defined constructs can shape the scope of inquiry and interpretation. Future studies should incorporate the perspectives of patients and caregivers to provide a more holistic understanding of frailty pathway implementation.

## Conclusion

This study highlighted the key factors that could shape the implementation of a frailty pathway in a tertiary acute care hospital. Healthcare professionals recognised the potential of a frailty pathway for older adults but cited barriers in the hospital context, including limited resources, workflow constraints, and the complicating presence of delirium. We found that socioeconomic disparities and cultural beliefs can further influence the adoption of frailty pathways. Addressing these challenges will require targeted education efforts, cultural shifts, multidisciplinary collaboration, and integrating of frailty processes into existing workflows (for example, embedding processes within clinical IT systems and standardised protocols). Our findings provide actionable insights to inform practice and design strategies to implement a future frailty pathway in acute care.

## Supplementary Information

Supplementary Material 1.

Supplementary Material 2

## Data Availability

The datasets generated and/or analysed during the current study are not publicly available due to ethical restrictions but are available from the corresponding author on reasonable request.

## Abbreviations

CGH: Changi General Hospital
IB: Integrated Building
CFIR 2.0: Consolidated Framework for Implementation Research 2.0
