# Supplementary material for: Perceptions of frailty pathway implementation in an acute setting among healthcare professionals: a qualitative study using the CFIR 2.0
Source: BMC Geriatr. 2026 Jun 3;26:1023. doi: 10.1186/s12877-026-07683-5 (PMC13455301; doi:10.1186/s12877-026-07683-5)
Supplement: Supplementary file 1 [file 12877_2026_7683_MOESM1_ESM.docx]

**FOCUS DISCUSSION GROUP INTERVIEW SCRIPT**

**Welcome** and thank you for volunteering to take part in this focus group discussion. You have been asked to participate as your point of view is important. I realize you are busy, and I really appreciate your time.

**Anonymity:** Despite being taped; I would like to assure you that the discussion will be anonymous. The tapes will be kept safely in a locked facility until they are transcribed word for word, then they will be destroyed. The transcribed notes of the focus group will contain no information that would allow individual participants to be linked to specific statements. You should try to answer and comment as accurately and truthfully as possible. I and the other focus group participants would appreciate it if you would refrain from discussing the comments of other group members outside the focus group. If there are any questions or discussions that you do not wish to answer or participate in, you do not have to do so; however please try to answer and be as involved as possible.

**Ground rules**

- The most important rule is that only one person speaks at a time. There may be a temptation to jump in when someone is talking but please wait until they have finished.
- There are no right or wrong answers
- You do not have to speak in any particular order
- When you do have something to say, please do so. There are many of you in the group and it is important that I obtain the views of each of you
- You do not have to agree with the views of other people in the group
- Does anyone have any questions? (answers).
- OK, let’s begin

**Warm up**

First, I’d like everyone to introduce themselves. Can you tell us your name (pseudonym)? How are you feeling today?

1) What are the current processes for frailty screening or interventions?
   a. When should frailty screening be administered?
   b. What do you think can be done to improve the current way of identifying frail patients?
   c. Are there any specific dedicated areas or care pathways for frail patients?
   d. Who is responsible for providing frailty interventions?
   e. How can frailty interventions be incorporated into daily work process?
   f. How are caregivers identified and involved in this process?
2) Do you think of frailty interventions are beneficial for patients?
   a. What interventions or treatments can help to reverse or slow frailty progression?
3) How knowledgeable are you about how to undertake frailty interventions?
4) What are some challenges or problems that you face in managing frail patients in the ED?
a. What are other recommendations to improve the current process of managing frail patients in the ED?

Managing care transitions,

5) How do frail patients transition from admission into a ward?
   a. What are some of the risks and challenges you have encountered while the patient is transitioning to or from the ward?
   b. How do you feel these challenges can be addressed?

Managing patients in the ward environment

6) What are the challenges of managing frail patients in the ward?
   a. How adequate is the ward equipment and environment in taking care of frail patients?
   b. What resources or technologies would you feel should be provided to staff to better manage and care for frail patients?
7) Do the ward routines help frail patients maintain their function?
   a. What changes would you recommend to ward processes to support frail patients?
8) Where can you get information to help you care and support frail patients?

Interprofessional collaboration,

9) How do the staff work together in identifying and managing frail patients?
   a. Which other staff groups do you work with when managing frail patients?
   b. What are the challenges you have encountered when working together with other providers to manage frail patients?
   c. How do you think such collaboration can be improved?

Managing patient discharge,

10) How effective do you feel the current processes are when it comes to safely discharge frail patients back to the community?
    a. How do teams involve NOK and family caregivers when discharging frail patients?
    b. How do you think this process can be improved?
11) What services can your teams offer frail patients after discharge?
    a. Is frailty training provided to caregivers and how can caregiver training be improved?

Staff education and training,

12) Do you feel sufficiently trained to handle frail patients?
    a. What training do you feel is most effective for learning how to care for frail patients?
13) Would you be willing to attend frailty training if available?
    a. How do you feel about following an instruction manual that will guide identification and management specifically for frail patients?

Thank you for your time!
